# Retraction: Integrating Epitaxial-Like Pb(Zr,Ti)O_3_ Thin-Film into Silicon for Next-Generation Ferroelectric Field-Effect Transistor

**DOI:** 10.1038/srep31300

**Published:** 2016-09-02

**Authors:** Jae Hyo Park, Hyung Yoon Kim, Gil Su Jang, Ki Hwan Seok, Hee Jae Chae, Sol Kyu Lee, Zohreh Kiaee, Seung Ki Joo

Scientific Reports 6: Article number: 2318910.1038/srep23189; published online: 03 23 2016; updated: 09 02 2016

This Article has been retracted by the authors. The data presented in Figures 1e, 1g, 1h, 2b-d, 4a-b, 4d-e, 6a-b and 7a-b were manipulated and are duplicated in other papers^1^,^2^,^3^,^4^,^5^.

All authors acknowledge these issues and agree to the retraction of the Article.
